# Integrative in silico and in vitro transcriptomics analysis revealed new lncRNAs related to intrinsic apoptotic genes in colorectal cancer

**DOI:** 10.1186/s12935-020-01633-w

**Published:** 2020-11-10

**Authors:** Fatemeh Akbari, Maryam Peymani, Ali Salehzadeh, Kamran Ghaedi

**Affiliations:** 1 Department of Biology, Faculty of Basic Sciences, Shahrekord Branch, Islamic Azad University, Shahrekord, Iran; 2 Department of Biology, Rasht Branch, Islamic Azad University, Rasht, Iran; 3 Department of Cell and Molecular Biology and Microbiology, Faculty of Biological Science and Technology, University of Isfahan, Isfahan, Iran

**Keywords:** Colorectal cancer, Apoptosis, lncRNAs, Meta-analysis, RNA sequence

## Abstract

**Background:**

Pathogenesis of colorectal cancer (CRC) is connected to deregulation of apoptosis while the effect of lncRNAs, as critical regulatory molecules, on this pathway is not clear well. The present study aimed to identify differential expression of genes and their related lncRNAs which are significantly associated with intrinsic apoptotic pathway in CRC.

**Methods:**

The connection between CRC and apoptosis was investigated by literature reviews and the genes were enriched by using Enrichr. At the next step, differential expression of enriched genes were evaluated between normal and tumor populations in data sets and were downloaded from GEO. Then, meta-analysis and probe re-annotation were performed. For lncRNAs selection through the highest expression correlation with each of candidate genes, mRNA-lncRNA interaction of screened genes and all of lncRNAs were visualized using Cytoscape. Identified differential expression genes and lncRNAs were validated using TCGA-COAD and the obtained data were confirmed by in vitro studies in the presence of Ag@Glu-TSC nanoparticle as an apoptotic inducer. Cytotoxicity and apoptosis induction effect of Ag@Glu-TSC on Caco-2 cells was determined via MTT and Annexin V/PI, respectively. The expression of genes and lncRNAs were assayed in presence of mentioned nanoparticle. Finally, the expression level of desired genes and lncRNAs were proven in CRC tissues compared to adjacent normal tissues.

**Results:**

After detection of 48 genes associated with intrinsic apoptosis in CRC according to literature, Enrichr screened 12 common genes involved in this pathway. Among them, 6 genes including *BCL2*, *BCL2L11*, *BAD*, *CASP7*, *CASP9*, and *CYCS* expression reduced in tumor tissue compared to normal according to meta-analysis studies and RNA-seq TCGA data. Afterwards, association of 8 lncRNAs comprising *CDKN2B-AS1*, *LOC102724156*, *HAGLR*, *ABCC13*, *LOC101929340*, *LINC00675*, *FAM120AOS*, *PDCD4-AS1* with more than 5 candidate genes were identified. In vitro studies revealed that four selected lncRNAs including, *CDKN2B-AS1*, *LOC102724156*, *HAGLR* and *FAM120AOS* were significantly increased in the presence of in optimum concentration of Ag@Glu/TSC and decreased in tumor tissues versus adjacent normal tissues.

**Conclusion:**

This study developed a new data mining method to screen differentially expressed lncRNAs which are involved in regulation of intrinsic apoptosis pathway in CRC quickly using published gene expression profiling microarrays. Moreover, we could validate a number of these regulators in the cellular and laboratory disease models.

## Background

Colorectal cancer (CRC) is the third and second common cancer among males and females, respectively [1, 2]. In 2018, around 1.8 million new cases and about 86,100 deaths were reported cause of CRC. The prevalence of CRC is predicted to increase by 60% in 2020 with 101 million deaths and 202 million new cases [3].

Apoptosis, as a well-known kind of cell death, plays an important role in multicellular systems such as cancer. In colorectal tissue, the imbalance between cell proliferation and apoptosis typically yields tumor growth. Although the molecular mechanisms of cell division and apoptosis are similar in normal and tumor cells, these mechanisms are aberrantly regulated in tumor cells [4]. Recently, modulating effect on the biological behaviors of tumor cells is of interest for researchers to focus on noncoding RNAs (NcRNAs). In the group of NcRNAs, long noncoding RNAs (lncRNAs) gained much attention thanks to their significant regulatory effects on carcinogenesis and tumor development [5]. Indeed, lncRNAs are well-introduced as oncogenes or tumor suppressor genes by modulating cell proliferation and apoptosis [6].

Since the effect of specific lncRNAs on apoptosis mechanisms in CRC progress have been less studied, here we investigate lncRNAs, related to apoptosis genes in CRC tissues using Gene Expression Omnibus (GEO) data. Subsequently, RNA sequences data from The Cancer Genome Atlas (TCGA) database were used to confirm the GEO data. The expression changes of candidate genes and lncRNAs in the Caco-2 cell line in presence of Ag@Glu-TSC nanoparticles as an apoptotic agent were analyzed. Then expression of these identified lncRNAs in CRC tissues were evaluated and compared with adjacent normal tissues.

## Materials and methods

### Data source and identification of differentially expressed genes

**Fig. 1:**
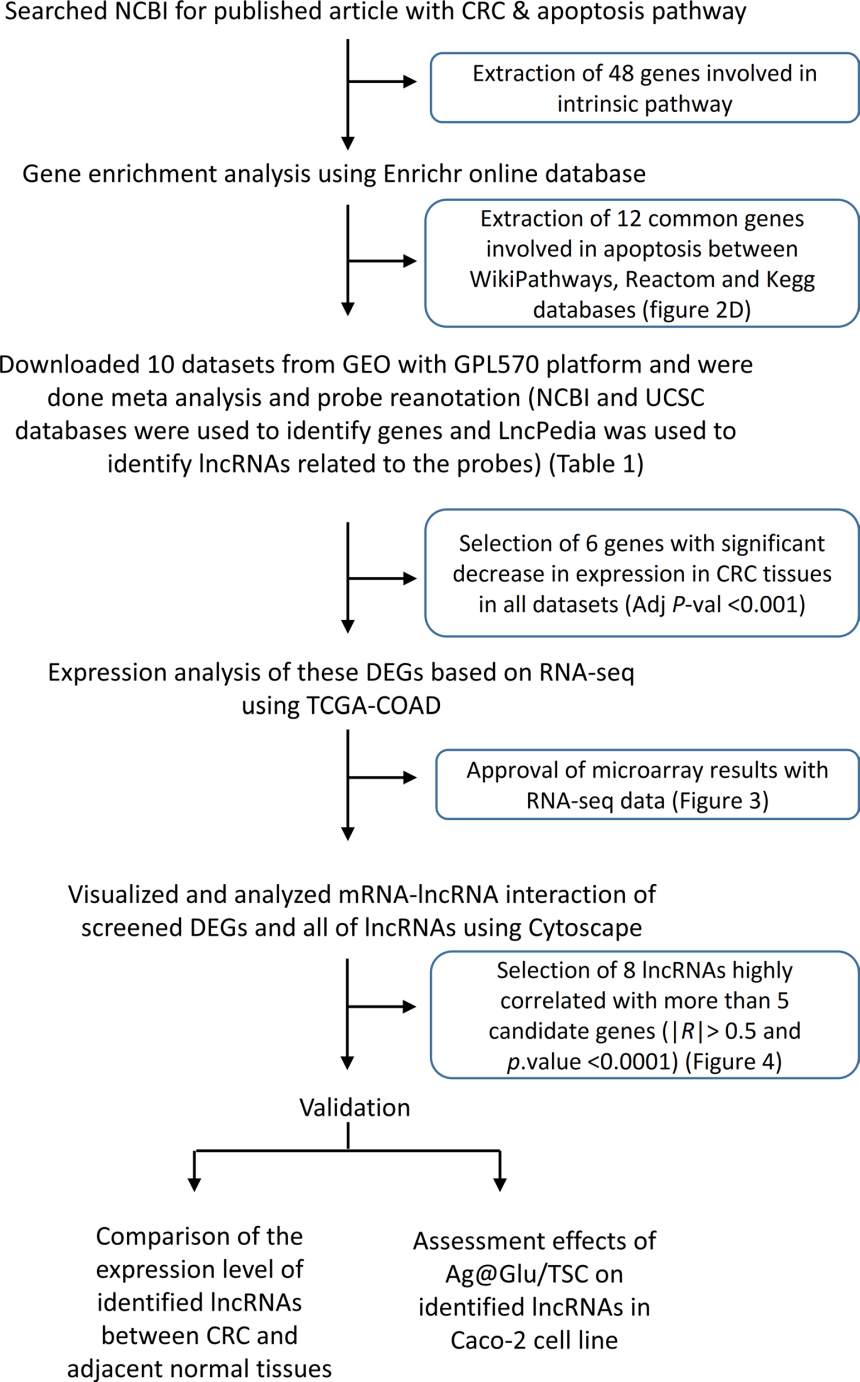
Study design flow chart. *DEGs* differential expression genes, *GEO* Gene Expression Omnibus, *KEGG* Kyoto Encyclopedia of Genes and Genomes, *TCGA-COAD* The Cancer Genome Atlas-Colorectal Adenocarcinoma, *lncRNAs* long non-coding RNAs, *adj P-val* adjusted *P*-value

A schematic process diagram of this study is presented in Fig. 1. The first important step in this study was selection of genes involved in intrinsic apoptosis in CRC based on literature review articles. Then gene enrichment was performed using Enrichr online database [7]. Selected genes from KEGG, WikiPathways and Reactome in Enrichr database were reported as genes involved in the internal apoptosis pathway with adjusted *P*-value < 0.05. The significantly enriched gene pathways terms (adjusted *P*-value < 0.05) were summarized and visualized using the REVIGO web [8]. Raw gene expression data of the GEO series (GSE) including GSE8671, GSE9348, GSE18105, GSE20916, GSE21510 and GSE37364 datasets were downloaded from the National Center of Biotechnology Information GEO database (https://www.ncbi.nlm.nih.gov/gds/). These studies include tumor samples at different stages and adjacent non-tumor samples. These samples were used for expression analysis of candidate apoptosis genes. All studies were normalized by the same method, RMA method form limma package [9] of R. Principal component analysis (PCA) was performed using the prcomp function in the built-in R stats package (*version* 3.2.2). The first two PCs were visualized using the ggbplot package (*version* 0.55). Subsequently, we performed hierarchical cluster analysis using the R package heatmaps. Then studies were merged through probes and used SVA package (ComBat), to eliminate the batch effect and performed a meta-analysis of ten CRC transcriptome datasets using the bioinformatics approach [10]. Of course, for this meta-analysis phase, we added four additional datasets that contained only tumor samples to the study population. Finally In this examination, we identified differentially expression genes (DEGs) from GEO datasets. The DEGs were selected out according to the criteria: |log (fold change)| < 1.5 and adj.*P*-val < 0.001. A summary information of these datasets was shown in Table 1. All studies selected for meta-analysis of the same technology as the Affymetrix Human Genome U133 plus 2.0 Array were named GPL570 because this platform is repeatedly used and includes 22,277 common probe sets [11]. NCBI and UCSC databases were used to find genes related to the probes used in these studies. Moreover, LncPedia was applied to detect probes related to lncRNAs [12]. To identify DEGs that are played key role in colon tumorigenesis, we employed an integrative analysis of TCGA colon adenocarcinoma (TCGA-COAD) using TCGA biolinks package [13]. The sample size for CRC available at TCGA includes 480 cancerous and 41 normal samples. Then obtained data were normalized by the DEseq2 package.

**Table 1:** Summary information of the datasets used in present study

Submission data	Country	Platform	Number of normal tissue	Number of tumor tissue	GEO accession
GSE4107	10	12	GPL570	Singapore	2006
GSE8671	32	32	GPL570	Switzerland	2007
GSE9348	12	70	GPL570	Singapore	2007
GSE18105	17	94	GPL570	Japan	2009
GSE21510	25	123	GPL570	Japan	2010
GSE22598	17	17	GPL570	Japan	2010
GSE23878	24	35	GPL570	Saudi Arabia	2010
GSE32323	17	17	GPL570	Japan	2011
GSE33113	6	90	GPL570	Netherlands	2011
GSE37364	38	56	GPL570	Hungary	2012
Total	198	546			

### Co-expression network construction and analysis

Map of mRNA-lncRNA interaction between DEGs and lncRNAs involved in apoptosis pathway, were visualized and analyzed using Cytoscape software (*version* 3.4) [14]. The yFile application were also used to layout and enhance the visual appearance. The Pearson correlation coefficient of DEG-lncRNA pairs was calculated according to their expression value. The co-expressed DEG-lncRNA pairs with the absolute value of Pearson correlation coefficient ≥ 0.5 were screened. Ultimately, lncRNAs were selected that were strongly correlated with the candidate genes in the intrinsic pathway.

### Synthesis of Ag@Glu-TSC nanoparticles

To prepare the desired nanoparticle, 100 mg glutamic acid was added to 50 ml of AgNo_3_ and stirred for 30 min (solution 1). Then for providing the solution 2, 20 mg of NaBH_4_ was dissolved in 50 ml of double distilled water and was added to solution 1 drop by drop and stirred for 3 h. The produced precipitate, defines as Ag@Glu, was washed with ethanol and double distilled water several time, and then dried in 40 °C for 24 h. Grafting the Thiosemicarbazide (TSC) on Ag@Glu was the next stage to generate Ag@Glu-TSC nanoparticle. For this stage, 60 ml ethanol was added to the achieved precipitate, and sonicated for 20 min. Then, 90 mg of TSC was added to the resulted solution, and it was stirred for 24 h. Ultimately, the final product was washed with ethanol and dried at 40 °C for 24 h [15].

### Characterization of synthesized Ag@Glu-TSC nanoparticles

The synthesized Ag@Glu-TSC nanoparticle was characterized using Fourier transform infrared (FTIR) spectroscopy (Shimadzu, Japan). Size assessment and morphological properties of the Ag@Glu-TSC nanoparticles were performed using transmission electron microscope (TEM) (Zeiss 100KV model, Germany) and scanning electron microscope (SEM) (*TESCAN MIRA3*, Czech Republic). Also, the data from X-ray diffraction (XRD) was measured using diffract meter (Philips X'Pert MPD, Netherlands). Finally, the energy-dispersive X-ray (EDX) spectrometry (TESCAN MIRA3, Czech Republic) was used to analyze the chemical composition of Ag@Glu-TSC nanoparticle.

### Cell culture

Caco-2 and HEK293 embryonic human *cell* lines as a colorectal cancer and a non-malignant cell line were obtained from National Cell Bank of Iran (Pasteur Institute, Tehran, Iran) and were used in this study. HEK293 and Caco-2 were cultured in Dulbecco’s modified Eagle’s medium (DMEM) or RPMI 1640 (Gibco, USA). These cell culture mediums were supplemented with 10% fetal bovine serum (FBS, Gibco, USA), penicillin (Gibco, USA) (100 µg/ml), streptomycin (Gibco, USA) (100 µg/ml), 2 mM l-glutamine. Cells were incubated in a humidified atmosphere 5% CO_2_ at 37 °C incubators.

### Cell viability assay

For evaluation of Ag@Glu-TSC nanoparticles cytotoxicity effect on Caco-2 and HEK293 cell lines, MTT assay 3-(4,5-dimethylthiazol-2yl)-2, 5-diphenyltetrazolium bromide (MTT) was performed. 1 × 10^4^ cells were cultured in each well of the 96-well plates, and they were incubated for 24 h in presence of different concentrations of Ag@Glu-TSC nanoparticles (5, 25, 31, 92, 125, 250, and 500 µg/ml). Then, 25 µg/ml MTT color solution was added to each well and after 4 h the solution over each well was removed and dimethyl sulfoxide (DMSO) was added to dissolve formazan crystals. At least, optical density at 570 nm was measured using an ELISA micro plate reader. The IC50 values of cells were calculated by the following formula:

$$ (\% )\;{\text{inhibition}} = \frac{{{\text{Abs}}\;{\text{of}}\;{\text{control}} - {\text{Abs}}\;{\text{of}}\;{\text{test}}}}{{{\text{Abs}}\;{\text{of}}\;{\text{control}}}} \times 100. $$

### Flow cytometry analysis of apoptosis/necrosis

Cells were permitted to adhere and proliferate for 24 h former to exposure to nanoparticle. The incubated cells were treated with IC50 concentration of Ag@Glu-TSC nanoparticles and maintained for an additional 24 h. The cells were separated from the wells due to treatment with trypsin and centrifugation, washed with PBS and then stained with 10 µl annexin-V-FLUOS and 5 µl propidium iodide (PI) based on the manufacturer protocol (Roch, Germany). The number of apoptotic and necrotic cells was assessed by flow cytometry device (Partec, Germany) and compared between treated and untreated Caco-2 cells.

### Sample collection, RNA extraction, cDNA synthesis and RT-qPCR

This study was approved by the Biomedical Ethics Committee of the Islamic Azad University of Shahrekord and also complies with the Ethics Code of IR.IAU.SHK.REC.1398.044. In the main-time all participants signed informed consent. All methods have been accomplished according to related protocols. Twenty tissue samples of intestinal section patients with CRC who were approved by a professional pathologist following Iran National Tumor Bank’s inspection and principles Clinical information of these samples was shown in Table 2 Also in this study, cultured cells were collected for expression of genes assay. Total RNA extraction from culture cells, tumor and adjacent normal colorectal tissue was performed with TRIzol (Sigma-Aldrich, Germany) kits, according to the manufacturer’s instruction. After determination of RNA quality, RNA samples were treated with *RNase*-free *DNase* I (Sinaclon, IRAN) according to protocol. cDNA synthesis was done by cDNA synthesis kit (Biofact, Korea). QPCR was done in Roche real-time PCR systems by using 10 µl SYBR Green PCR Master Mix (Takara, Japan), 10 pmol/µl of each primer, and 50 ng cDNA in a final volume of 20 µl for each reaction, Expression samples was normalized to *β-ACTIN* as an internal control. All measurements were carried out in triplicate and data were analyzed by ΔCt method. The specific primers for each mRNA and lncRNA which are listed in Table 3 were designed using Beacon Designer (https://www.ncbi.nlm.nih.gov/tools/primerblast/). (*version* 7.2, USA) obtained from Metabion Company (Germany) and confirmed with “NCBI Primer BLAST” (https://www.ncbi.nlm.nih.gov/tools/primerblast/).

**Table 2:** Patients’ demographic and clinical information (n = 20)

Variables	Number
Age	
< 50	16
≥ 50	4
Sex	
Male	16
Female	4
TNM stage	
I	2
II	8
III	8
IV	2
Grade	
I	4
II	11
III	2
IV	2
Unknown	1
Tumor size (cm)	
< 5	6
> 5	14
Smoking	
Smoker	3
Non-smoker	17

**Table 3:** The specific primer sequences for selected lncRNAs and mRNAs

Gene name	Primer sequence (5′ to > 3′)	Annealing (°C)	Length product (bp)
HAGLR (lnc-AC009336.1-2)	CAGACTCAGCAGATACTC TCATCTCCTCTTCCTACC	62	204
CDKN2B-AS1 (lnc-MTAP-1)	TATTCCTGGCTCCCCTCGTC TGACCTCGCTTTCCTTTCTTCC	61	185
PDCD4-AS1 (lnc-BBIP1-1)	GTCCTACCTCCCCCACTAAC TGGAACAATCCCTCCCACA	58	181
LINC00675 (lnc-PIRT-1)	TTAGAACCAACCACAAGCACCA AGCCAGTGAGGAGAAATAGCAAC	60	88
FAM120AOS (nc-FAM120AOS-4)	GCAGAACACCAAAGGAGACC ATTTTTGCATCAGCCCAAAG	60	195
LOC101929340 (lnc-NRGN-1)	TCAACCAACAGGCATCAGAA CCGCAAATCCAGGTAAGAAC	58	143
ABCC13 (lnc-RBM11-5)	AGGAATCAAGAGAGGCAAAAAGC GAGTGGGCTAGTGAAGGACAA	59	162
LOC102724156 (lnc-CDK20-8)	GGACACCCTAGAGGCAGAGA TCAAACTTGTGTGCTGAAGAGA	62	150
CYCS	GGCGGCTGTGTAAGAGTATCCA AACCTTACCCCAGTGGTGCTC	60	192
CASP9	AACCTTACCCCAGTGGTGCTC ATCTGCATTTCCCCTCAAACTCTCA	62	138
BCL2	AACATCGCCCTGTGGATGACT GGCAGGCATGTTGACTTCACTT	62	223
CASP7	GGACCGAGTGCCCACTTATC TCGCTTTGTCGAAGTTCTTGTT	60	89
BAD	GGAGGATGAGTGACGAGTTTG TTCGGGATGTGGAGCGAAG	60	184
GAPDH	TGAAGTCGCAGGAGACAACC TGCCGCCTGGAGAAACC	60	121

### Statistical analysis

All data were expressed (mentioned) as the mean ± standard deviation (SD). Statistical analysis and curve fitting were carried out (accomplished) by using SPSS 20.0 and GraphPad Prism 8.0 (GraphPad Software, San Diego, CA). Based on the test condition, Student's t-tests or Mann–Whitney U-test were used to determine the differences between desired groups. All the data were considered significant when the *P* < 0.05 and with a 95% corresponding confidence level.

## Results

### Preparation of a gene list involve in intrinsic apoptosis pathway in CRC

**Fig. 2:**
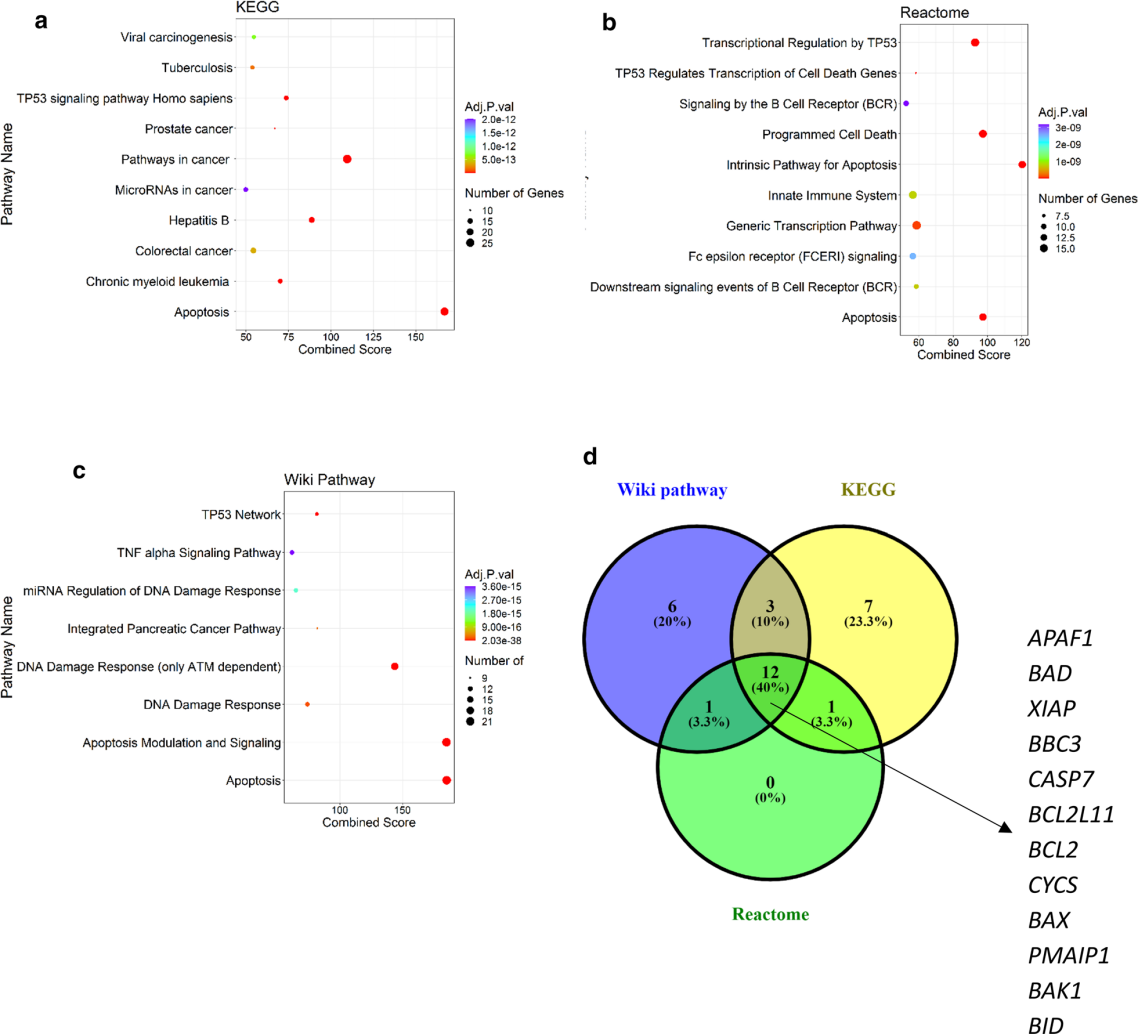
Pathway enrichment analyses of screened genes in Enrichr database. Significantly enriched pathways of screened genes using the REVIGO web based on **a** KEGG, **b** reactome and **c** WikiPathways database. **d** Venn diagram generated with Venny 2.1 showing the proteins annotated for the intrinsic apoptosis signaling pathway in three different databases. 12 of these genes are common to all databases (40%)

Based on literature review [16–31], 48 genes were identified in related to the apoptosis pathway. This gene list is shown in Additional file 1: Table S1. We further examined the putative biological function of these genes by using pathway enrichment analysis (Fig. 2a–c). Our data revealed that these genes were significantly enriched in apoptosis pathway. The list of overlapped genes from all three databases, as important genes in desire pathway in CRC, are shown in the Fig. 2d. Moreover, the results of the Enrichr analysis are summarized in Table 4. Most of these enriched pathways are related to apoptosis. These identified pathways are downstream or upstream of the apoptotic pathway and the main result of these pathways is cell death.

**Table 4:** Summarized of the Enrichr analysis results

Database	Gene names
KEGG pathway	HRK-APAF1-BCL2A1-BAD-XIAP-FOS-NFKBI-RELA-BBC3-CASPS9-NRAS-CASP7-BCL2L11-AKT3-BCL2-CYCS-BAX-PMAIP1-FAS-BAK1-RAFI-BID-HRAS
WikiPathways	HRK-APAF1-BCL2A-CDKN2A-BIK-BAD-XIAP-FOS-NFKB1-BBC3-CASP9-CASP7-BCL2L11-BCL2-CYCS-BAX-PMAIP1-FAS-BAK1-BID-BCL2L2-BOK
Reactome pathway	APAF1-BAD-XIAP-BBC3-CASP9-CASP7-BCL2L11-AKT3-BCL2-CYCS-BAX-PMAIP1-BAK1-BID
Overlapped genes of databases	APAF1-BAD-XIAP-BBC3-CASP7-BCL2L11-BCL2-CYCS-BAX-PMAIP1-BAK1-BID

### Expression trends of selected genes in GEO is as similar as TCGA database

**Fig. 3:**
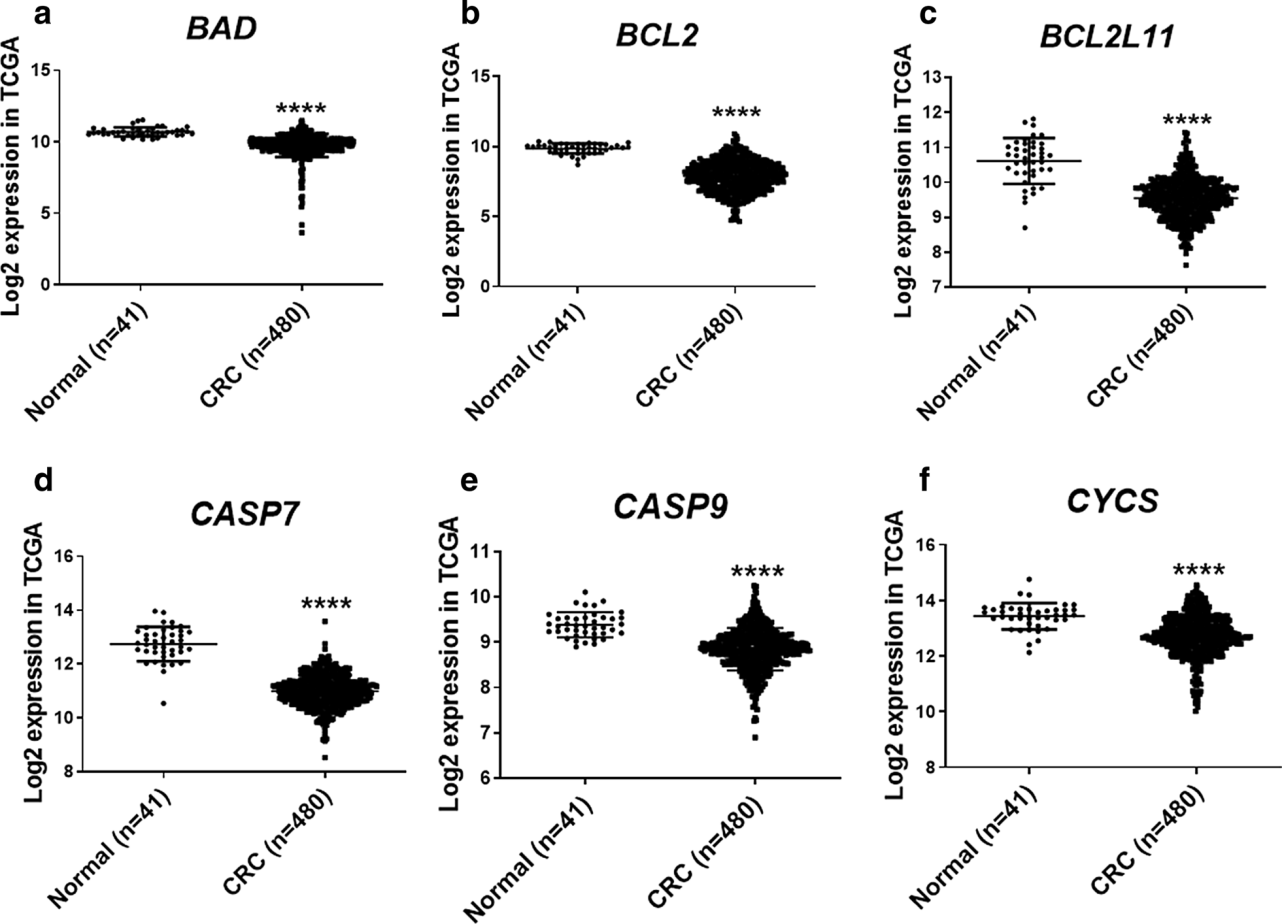
Analysis of candidate genes expression in The Cancer Genome Atlas (TCGA). **a**–**f** The Cancer Genome Atlas (TCGA) database was utilized to analyze to genes expression in 480 patients with CRC in TCGA RNA sequence and were compared to 41 normal samples. ****P value < 0.0001

As shown in Additional file 2: Figure S1A–F, a total of 6 GEO series (GSE) in the GEO database were elicited for the present study according to the inclusion principle. By utilizing heatmap and PCA, the quality control results of two studies from chosen datasets were obtained (Additional file 3: Figure S2A–D). The PCA results were appropriate for distinguishing between tumor and normal tissue samples. We evaluated the expression changes of candidate genes between CRC and normal tissue by the meta-analysis of 6 GEO datasets. Then the genes were selected that their expression levels were significantly down regulated in all cancerous tissues compared with adjacent non-tumor tissues with adj.*P*-val < 0.001. Finally, these analysis showed that six genes including *BCL2*, *BCL2L11*, *BAD*, *CASP7*, *CASP9*, and *CYCS* significantly had a reduced expression level in all these datasets. The accuracy confirmation of candidate genes in meta-analysis was examined by performing analysis of RNA-seq data available at TCGA database. As shown in Fig. 3a–f, the expression of the nominate genes between CRC tumor samples and normal colon tissue was assayed in the RNA-seq data and these data confirmed the microarray results.

### Co-expression network analysis of differently expressed lncRNAs and mRNAs in intrinsic apoptosis pathway

**Fig. 4:**
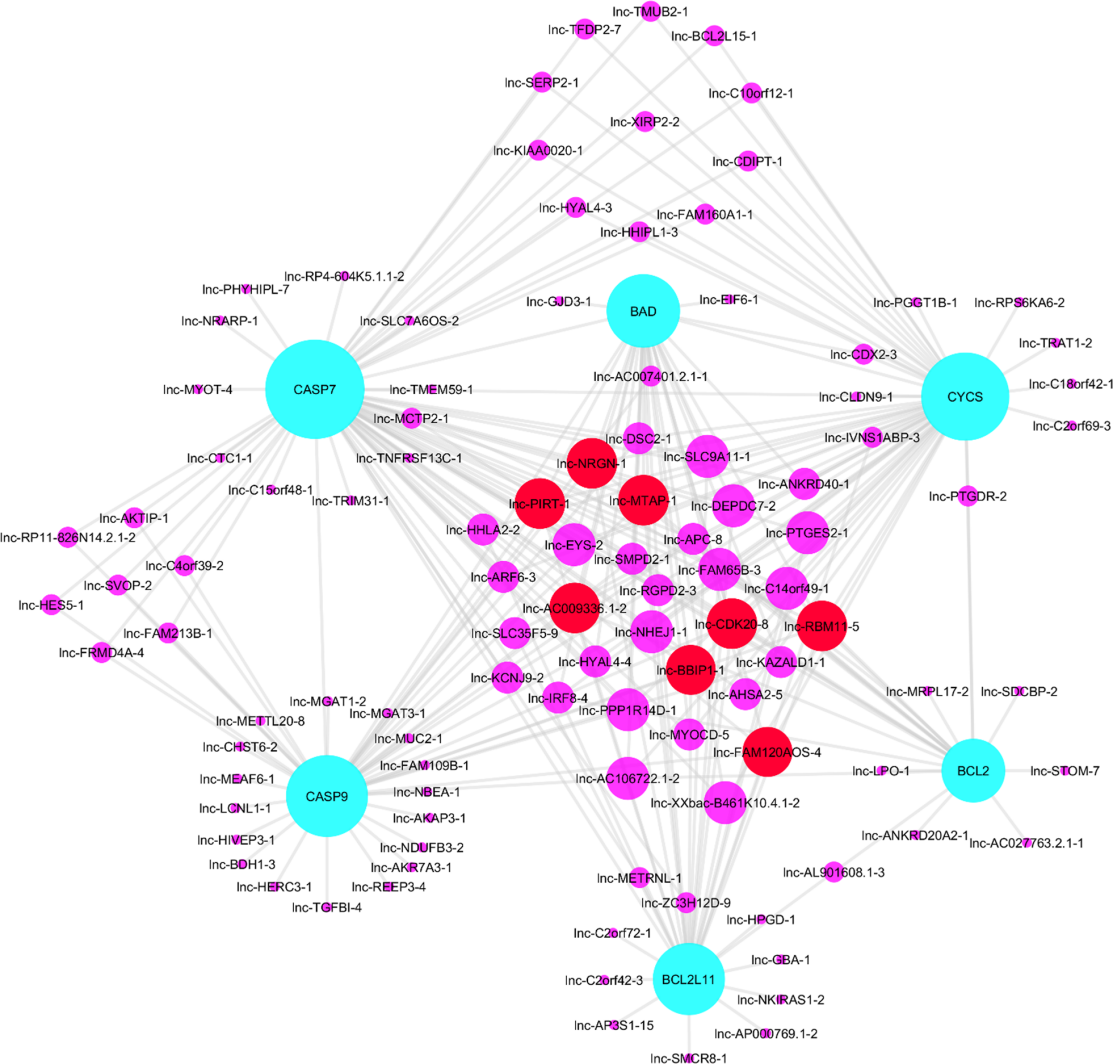
Construction of lncRNA-mRNA co-expression networks. LncRNA-mRNA co-expression networks of all 105 differentially expressed lncRNAs and 6 mRNAs with |Pearson correlation coefficient ≥ 0.5 and *P* value < 0.0001|. Red circles represents lncRNA with highly correlation and purple represents lncRNAs with poor correlation with candidate mRNAs. *lncRNA* long non-coding RNA

Pearson correlation coefficient was calculated between each candidate gene in apoptotic pathway (including BCL2, BCL2L11, BAD, CASP7, CASP9 and CYCS) and all lncRNAs in GPL570 chip. For this phase of the meta- analysis study, four additional datasets containing only tumor colorectal samples were added to the study. The lncRNAs with the highest expression correlation with each of the candidate genes (|R|> 0.5 and *P* value < 0.0001) were selected for co-expression network construction. The network of apoptotic pathway genes that are reduced in cancer tissue and the lncRNAs that have a significant expression relation with these genes, are displayed in the Fig. 3. As shown in Fig. 4, a total of 105 lncRNAs and 6 candidate mRNAs were included in this network. Among them, *CDKN2B-AS1*, *LOC102724156*, *HAGLR*, *ABCC13*, *LOC101929340*, *LINC00675*, *FAM120AOS* and *PDCD4-AS1* were identified as key lncRNAs in this network which associated with more than 5 candidate genes and were presented in Additional file 4: Table S2.

### Characterization of Ag@Glu-TSC

**Fig. 5:**
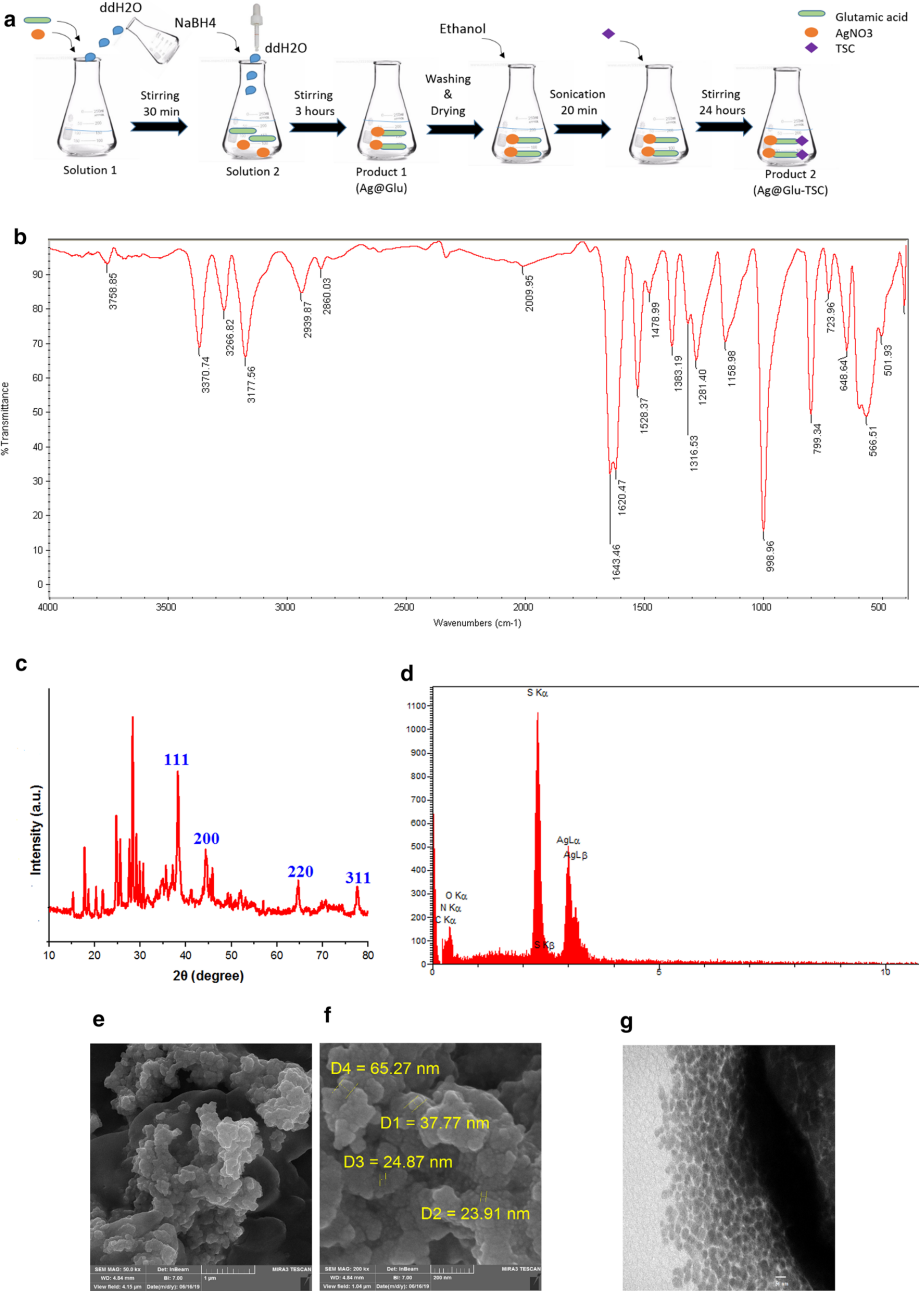
Synthesis and characterization of Ag@Glu-TSC. **a** Schematic diagram of the synthesis of Ag@Glu-TSC. **b** FTIR picks of nanoparticle. **c** XRD pattern of silver nanoparticles synthesized. **d** EDX spectrum of synthesized silver nanoparticles. **e**, **f** SEM images of nanoparticle. **g** TEM image of nanoparticle. *FTIR* Fourier-transform infrared spectroscopy, *XRD* x-ray diffraction, *EDX* energy dispersive x-ray, *SEM* scanning electron microscopy, *TEM* transmission electron microscopy

The synthesis process of Ag@Glu-TSC nanoparticles is illustrated in Fig. 5a. First of all, functionalization of Ag nanoparticles was done with glutamic acid. The produced nanoparticles (Ag@GA) were then conjugated with bio-reactive TSC moiety through covalent bonding of terminal amino groups of TSC and carboxylic groups of the glutamic acid. FTIR spectrum of synthesized Ag@Glu-TSC nanoparticles is exhibited in Fig. 5b. Robust bands at 3370 and 1640 cm^−1^ present the vibrational modes of water molecules adsorbed on the surface of the synthesized materials. The bands at about 2939 and 2860 cm^−1^ can be referred to the asymmetric CH_2_ stretching bands [32]. The bands at around the 800 can be recognized to the cm^−1^ m(C=S) and the band observed at 1281 cm^−1^ can be attributed to the m(C–N) [33]. Besides, the amide formation was proved with an amide bending vibration at around 1528 cm^−1^. This pick suggests the TSC attachment on the Ag@Glu nanoparticles, is formed via amide connection between GA and TSC. The crystal structure of synthesized nanoparticle was determined using XRD (Fig. 5c). The peaks of Ag@Glu-TSC nanoparticles in 2θ degree 38.28°, 44.04°, 64.34° and 77.28° correspond to (111), (200), (220) and (311) Bragg’s reflection of Ag (JCPDS card no. 04-0783). The sharp peaks had a good match with JCPDS (08-0726) file for TSC. Moreover, high intensity and sharpness of XRD peaks show great quality of the synthesized nanoparticles. The presence of silver and other compositional elements in the final structure were confirmed by using EDX spectroscopy (Fig. 5d). The elements of O, N, C, K, and S were characterized in synthesized nanoparticle (Table 5). Moreover, the morphology and size of Ag@Glu-TSC nanoparticles were evaluated using electron microscopy. SEM image of manufactured nanoparticles depicts a spherical shape according to Fig. 5e, f. Additionally, the TEM image, measured the mean size of the Ag@Glu-TSC nanoparticles approximately 50 nm (Fig. 5g).

**Table 5:** Results of EDX analysis of Ag@Glu-TSC nanoparticles

Elements	K	Kr	Weight %	Atomic %	ZAF
C	0.0831	0.0404	16.84	27.59	0.2399
N	0.1923	0.0934	40.73	57.25	0.2293
O	0.0000	0.0000	0.00	0.00	0.1270
S	0.3283	0.1595	17.19	10.55	0.9280
Ag	0.3963	0.1925	25.24	4.61	0.7628
Total	1.0000	0.4859	100.00	100.00	

### Apoptosis induction in presence of Ag@Glu-TS nanoparticles

**Fig. 6:**
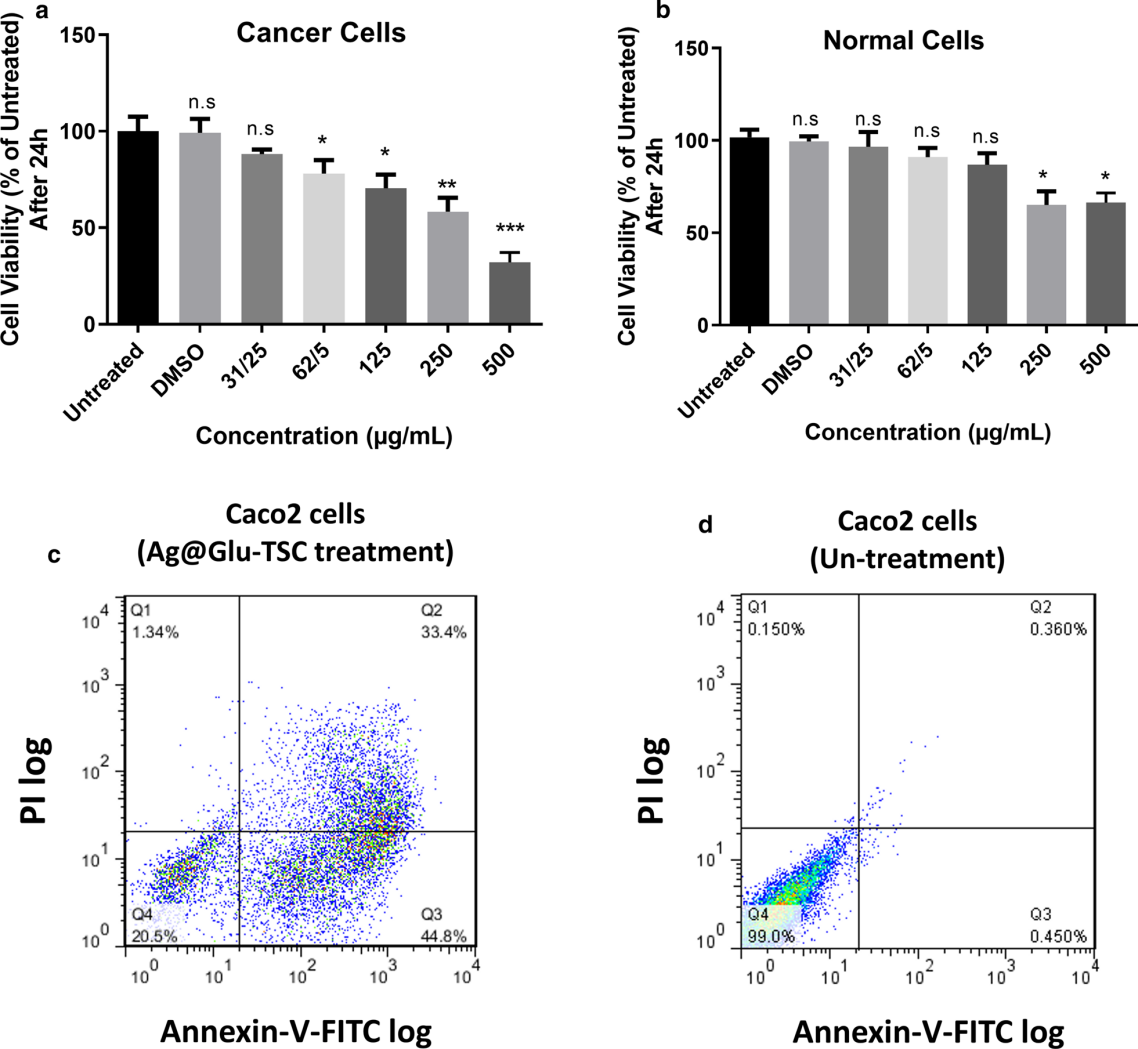
Direct effects of Ag@Glu-TSC on cell viability and apoptosis of Caco2 cells. **a** Dose-dependent effects of nanoparticles on cancer cells viability and normal cells after 24 h. **b** Comparison of nanoparticle administration with 247.22 µg/ml on the apoptosis in Caco-2 cells between treated and untreated group using flow cytometry. *P value < 0.05, **P value < 0.01 and ****P value < 0.0001

Cellular toxicity of various concentrations of Ag@Glu-TSC nanoparticles on Caco-2 and non-malignant HEK-293 cell lines was assessed via MTT assay technique. The concentration of 500 µg/ml of Ag@Glu-TSC nanoparticles showed the maximum cell growth inhibition in Caco-2 cells (*P* < 0.001). IC50 nanoparticle concentration was calculated as 247.22 and 380 µg/ml for Caco-2 and HEK-293 cells, respectively, (Fig. 6a, b).

Moreover, dot plots of Annexin V/PI staining are shown in Fig. 6c, d. Exposure of Caco-2 cells with IC50 concentration of Ag@Glu-TSC exhibited about 44.8% early stage of apoptosis and 33.4% late stage of apoptosis. In comparison, 0.45% of untreated cells were in early stage of apoptosis and 0.36% in late stage of apoptosis (Fig. 6c, d).

### *CDKN2B-AS1, LOC102724156, HAGLR* and *FAM120AOS* as selected lncRNAs up-regulated as well as *BAD*,* CASP9* and *CYCS* in cancer cell and tissues

**Fig. 7:**
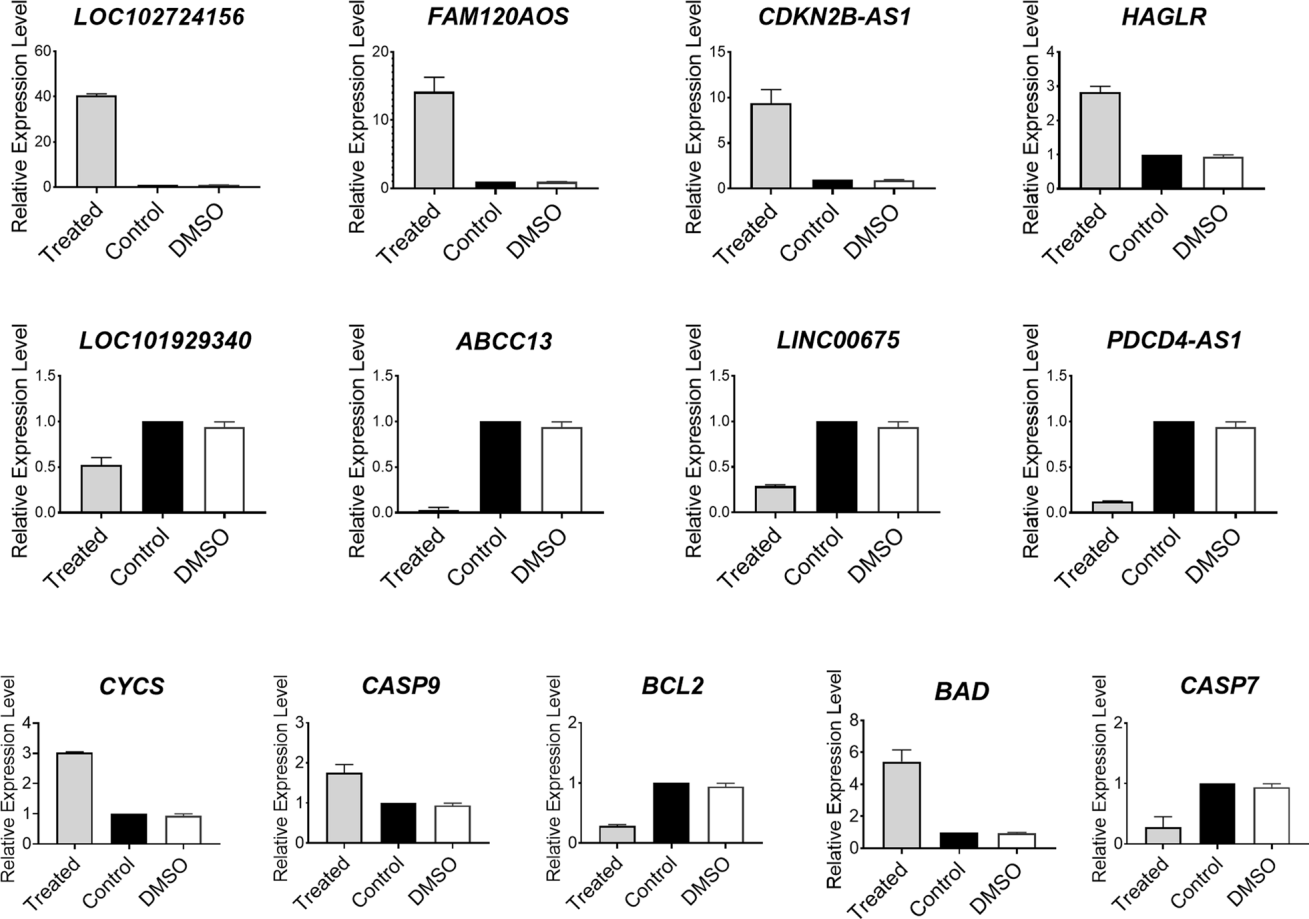
Relative expression level of candidate genes and lncRNAs involved in intrinsic pathway in presence of Ag@Glu-TSC. Expression level of relative genes and lncRNAs after treating by Ag@Glu-TSC for 24 h by RT-qPCR

In order to evaluate alterations in candidate genes and lncRNAs expression, RT-qPCR was performed, representing that *BAD*, *CYCS*, *CASP9*, *CDKN2B-AS1*, *LOC102724156*, *HAGLR* and *FAM120AOS* levels were significantly increased following treatment with 247.22 µg/ml Ag@Glu-TS for 24 h, in comparison with untreated cells and DMSO treatment as control groups (Fig. 7).

**Fig. 8:**
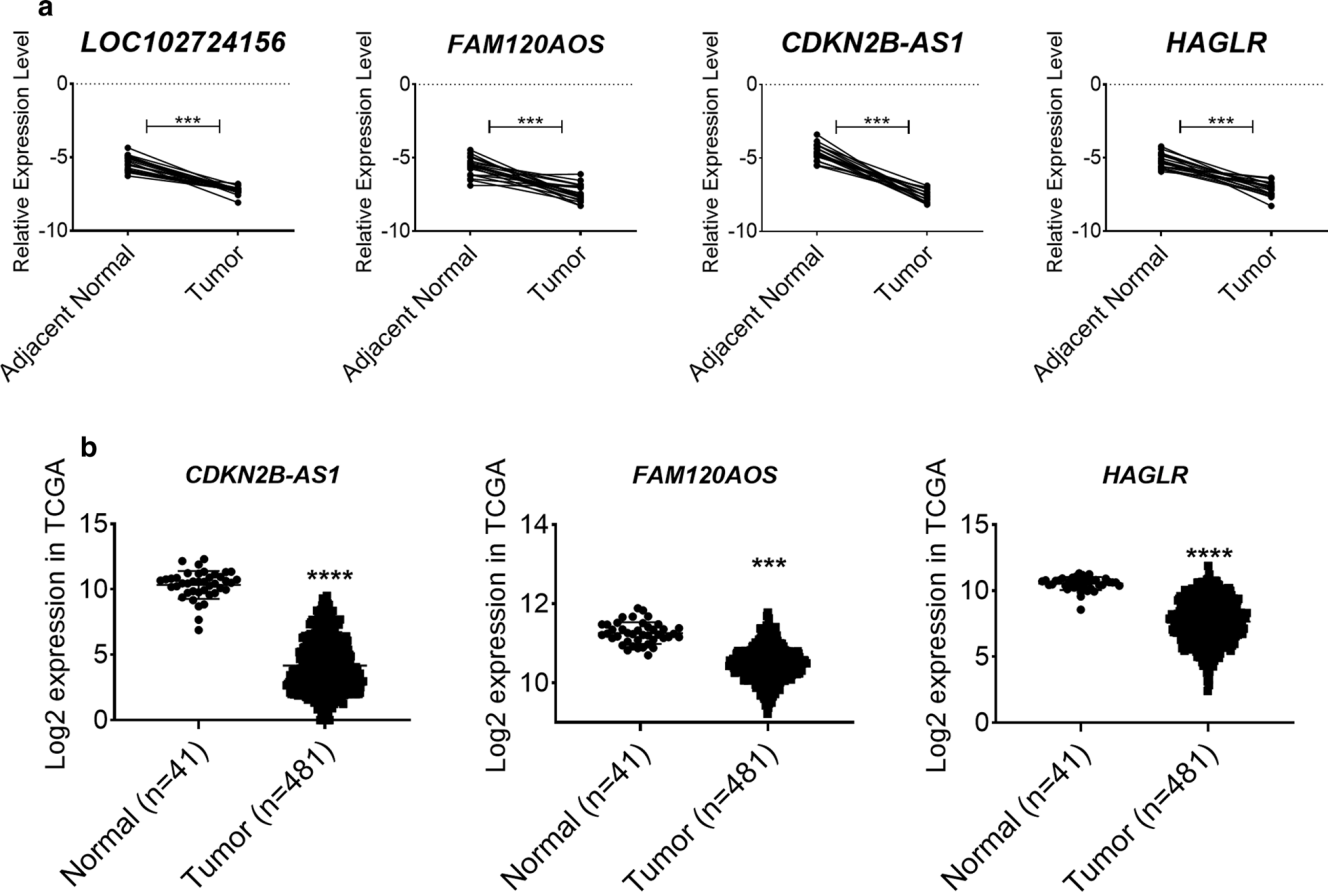
Validation of the selected lncRNAs using cancer and adjacent normal tissues and TCGA-COA dataset. **a** Comparative expression level of lncRNAs between tumor and adjacent normal tissue groups by RT-qPCR. **b** Expression analysis of extracted LncRNAs from TCGA database in tumoral and normal tissues in log_2_ scale. ***P value < 0.001 and ****P value < 0.0001

Since expression patterns of four lncRNAs and three candidate genes were similar in cellular model, we hypothesized that lncRNAs expression is altered in tumor tissues. As depicted in Fig. 8a, the expression level of mentioned lncRNAs decreased in tumor tissues versus adjacent normal tissues CRC patients significantly and confirmed data of cell culture model. Moreover, expression data of *CDKN2B-AS1*, *HAGLR* and *FAM120AOS* were validated with RNA sequence data obtained TCGA source (Fig. 8b) however there was not any expression data about *LOC102724156* in this database.

## Discussion

Apoptosis is a form of programmed cell death that leads to the elimination of damaged and unwanted cells and has a critical function in both normal and pathological processes [34]. Apoptotic cells show various biochemical modifications such as DNA and protein breakdown, caspase activation, membrane blebbing and phagocytic recognition that together result in the distinctive structural pathology [35]. Activation of a group of cysteine proteases called “caspases “appears to be important during apoptosis mechanism. Caspases have been widely considered by their known acts in apoptosis (caspase-3, -6, -7, -8, and -9 in mammals) and among them, Caspase 8 is known as an initiator caspase which is able to activate the other caspases [36]. Most apoptotic programs fall into either the extrinsic or intrinsic class. Noticeably, intrinsic apoptosis is also known as mitochondrial apoptosis because it depends on factors released from the mitochondria and can active by a vast array of cellular stresses and developmental signals that finally results in cytochrome c release from the mitochondria and apoptosome formation, subsequently. The extrinsic apoptosis route is activated through the binding of a ligand to a death receptor, which in turn leads, under the support of the adapter proteins (FADD/TRADD), to recruitment, dimerization, and activation of caspase-8. At the next step, active caspase-8 either initiates apoptosis directly by cleaving and through activating executioner caspase (-3, -6, -7), or activates the intrinsic apoptotic pathway thereby cleavage of BID to create effective cell death [37]. This process is rigidly controlled by BCL-2 family proteins. BCL-2 proteins classify into two groups: The pro-apoptotic proteins including: BAX, BAK and BAD, BCL-XS, BID, BIK, BIM and HRK and the anti-apoptotic proteins containing: BCL-2, BCL-XL, BCL-W, BF1-1 and MCF-1 [38]. Although it has been demonstrated that apoptosis can prevent of tumor progression, there is a wide range of means to inhibit this kind of cell death. Tumor-suppressor genes and oncogenes are commonly associated with apoptosis and CRC development. It appears that all routine remedies mostly operate via intrinsic apoptosis pathway and drug resistance can be the result of the failure of this way [39]. Till now, all methods which target the intrinsic apoptosis pathway were focused on BCL-2 family proteins. The current study aimed to identify DEGs that are significantly associated with the apoptotic pathway, and exploring the related lncRNAs at the whole genome level characteristic of CRC. lncRNAs have been established as useful biomarkers in CRC, and may be effective in early cancer detection and inhibition of CRC developing into malignancies. Furthermore, they may also act in early treatment. In addition the detection of single lncRNA alterations by PCR-based methods, genome-wide analysis can be accomplished by RNA-Seq and microarrays. Convinced lncRNAs connected to apoptotic pathway were previously analyzed in colorectal diseases. However, the main attention of previous studies was limited to one or two lncRNAs [40]. In this research, for the first time, we could identify putative lncRNAs involved in regulation of intrinsic apoptosis genes by insertion of CRC cases in a genome-wide lncRNA expression analysis. In this regard, we investigated all CRC expression profiling data in GEO database. At the next step, ten microarrays were selected, which 6 of them were included CRC cases and normal tissues, and 4 datasets were contained CRC tissues only. Based on datasets Meta-analysis in the current study, 105 candidate lncRNAs were differentially expressed along the CRC in related to 6 genes of intrinsic apoptosis pathway that could be validated on independent microarray results and TCGA COAD dataset. Among these 105 lncRNAs, *CDKN2B-AS1*, *LOC102724156*, *HAGLR*, *ABCC13*, *LOC101929340*, *LINC00675*, *FAM120AOS*, *PLEKHA7* and *PDCD4-AS1* were recognized as key lncRNAs in this network which are associated with more than 5 candidate genes according to co-expression network analysis using cytoscape. Although, comprehensive analysis of large‐scale samples for evaluating of the differentially expressed apoptosis genes and lncRNAs in CRC patients were performed, it seems that these findings need to be evaluated in CRC tissues and in vitro cell model. For this purpose, Caco-2 cell line was selected and Ag@Glu-TSC was used as an apoptosis inducer agent in these cells on the one hand, Shandiz et al. [15] showed the novel Ag@Glu-TSC nanoparticles are able to inhibit the proliferation of MCF7 cancer cell line. In the present paper the apoptotic effect of Ag@Glu-TSC on Caco-2 cell line was proved and the expression of *BAD* and *CYCS* were significantly increased after treatment with this nanoparticle. On the other hand, *CDKN2B-AS1*, *LOC102724156*, *HAGLR* and *FAM120AOS* expression levels were significantly increased in the presence of the aforementioned nanoparticle. After expression level assessment of these 4 lncRNAs in cancer tissue, we founded a significant decreased expression in tumor tissues compared to adjacent normal tissue. Alsibai et al. identified a high positive correlation between levels of expression of *CDKN2B-AS1*and three tumor suppressor genes. Indeed, they showed an increased expression of, *CDKN2B-AS1* in invasive colon carcinoma compared to normal tissues [41]. So far, there is no published papers about lncRNAs which are recognized in our study.

## Conclusion

We have used a new data mining method to screen quickly differentially expressed lncRNAs involved in regulation of intrinsic apoptosis pathway in CRC using published gene expression profiling microarrays. Moreover, in vitro assessments were performed to validate and confirm some of these differentially expressed lncRNAs. The present study has successfully demonstrated that the identified lncRNAs are potential therapeutic targets in CRC through targeting some intrinsic apoptosis pathway genes.

## Supplementary information

**Additional file 1: Table S1.** List of 48 genes in related to the apoptosis pathway.

**Additional file 2: Figure S1.** Volcano plot of differential expressed genes (DEGs) between CRC and normal samples from different GSEs based on adj.*p*-value and log_2_ (fold-change) at level of 0.0001 and logFC cutoff of 0.5. Colored dots correspond to individual genes whose expression differences were significant based on both adj.*p*-value and logFC value (red dots), only *p*-value (blue dots), only logFC (blue dots), or not significant (green dots) in either.

**Additional file 3: Figure S2.** The quality control results of two dataset GSE8671 and GSE9348 by utilizing PCA and heatmap.

**Additional file 4: Table S2.** LncRNAs list with the highest expression correlation with each of the candidate genes.

## Data Availability

Data sharing not applicable to this article as no data-sets were generated or analyzed during the current study.

## Abbreviations

MTT: 3-(4,5-Dimethylthiazol-2yl)-2,5-diphenyltetrazolium bromide
CRC: Colorectal cancer
DEGs: Differentially expression genes
DMSO: Dimethyl sulfoxide
DMEM: Dulbecco’s Modified Eagle’s Medium
EDX: Energy-dispersive x-ray
FBS: Fetal bovine serum
GEO: Gene Expression Omnibus
GSE: GEO series
lncRNAs: Long noncoding RNAs
NcRNAs: Noncoding RNAs
PCA: Principal component analysis
PI: Propidium iodide
SD: Standard deviation
SEM: Scanning electron microscope
TCGA: The Cancer Genome Atlas
TEM: Transmission electron microscope
TSC: Thiosemicarbazide
XRD: X-ray diffraction
