# Supplementary material for: Integrative in silico and in vitro transcriptomics analysis revealed new lncRNAs related to intrinsic apoptotic genes in colorectal cancer
Source: Cancer Cell Int. 2020 Nov 10;20:546. doi: 10.1186/s12935-020-01633-w (PMC7653898; doi:10.1186/s12935-020-01633-w)
Supplement: Supplementary file 1 — Additional file 1: Table S1. List of 48 genes in related to the apoptosis pathway. [file 12935_2020_1633_MOESM1_ESM.docx]

Table S1: List of 48 genes in related to the apoptosis pathway.

| Ref Number | Database | Gene Names |
| --- | --- | --- |
| 16 | Banck *et al*., 2013 | Bcl-2,bcl-xl,BCL2L2,BCL-XL,BCL-W,BCL3,BCL6,BCL10,BCL1,BCL9,Bax, Bak, Bid, Bad, Bok,BAK1,BBC3,BIK,BNIP3L,HRK |
| 17 | Suzuki *et al*., 2004 | CASPS3,CASP7,CASP9 |
| 18 | [Farrow](https://www.sciencedirect.com/science/article/abs/pii/S0959437X9690009X#!) *et al*.,2013 | Mcl-1,A1 |
| 19 | Nikki *et al*., 2019 | HRAS, RAS, NRAS, RAF1 |
| 20 | Moorchung *et al*., 2014 | NFKB2, NFKB1 |
| 21 | Vlahopoulos *et al*., 2019 | RELA,RELB |
| 22 | Hou *et al*., 2019 | MDM2,MDM4 |
| 23 | Wang *et al.*, 2016 | ABL1,ABL2 |
| 24 | Kaori *et al*., 2011 | CDKN1A,CDKN2A |
| 25 | Hartman *et al*., 2020 | FAS |
| 26 | Atri, *et al.*, 1998 | MLH1,XIAP |
| 27 | Yang *et al*., 2014 | CYCS |
| 28 | Yifeng*et al*., 2018 | AKT3 |
| 29 | Wee *et al*., 2008 | HIPK2 |
| 30 | Jansson *et al*., 2003 | PMAIP1 |
| 31 | Acehan *et al*., 2002 | APAF1 |
