# Supplementary figures and images for: Integrative in silico and in vitro transcriptomics analysis revealed new lncRNAs related to intrinsic apoptotic genes in colorectal cancer
Source: Cancer Cell Int. 2020 Nov 10;20:546. doi: 10.1186/s12935-020-01633-w (PMC7653898; doi:10.1186/s12935-020-01633-w)

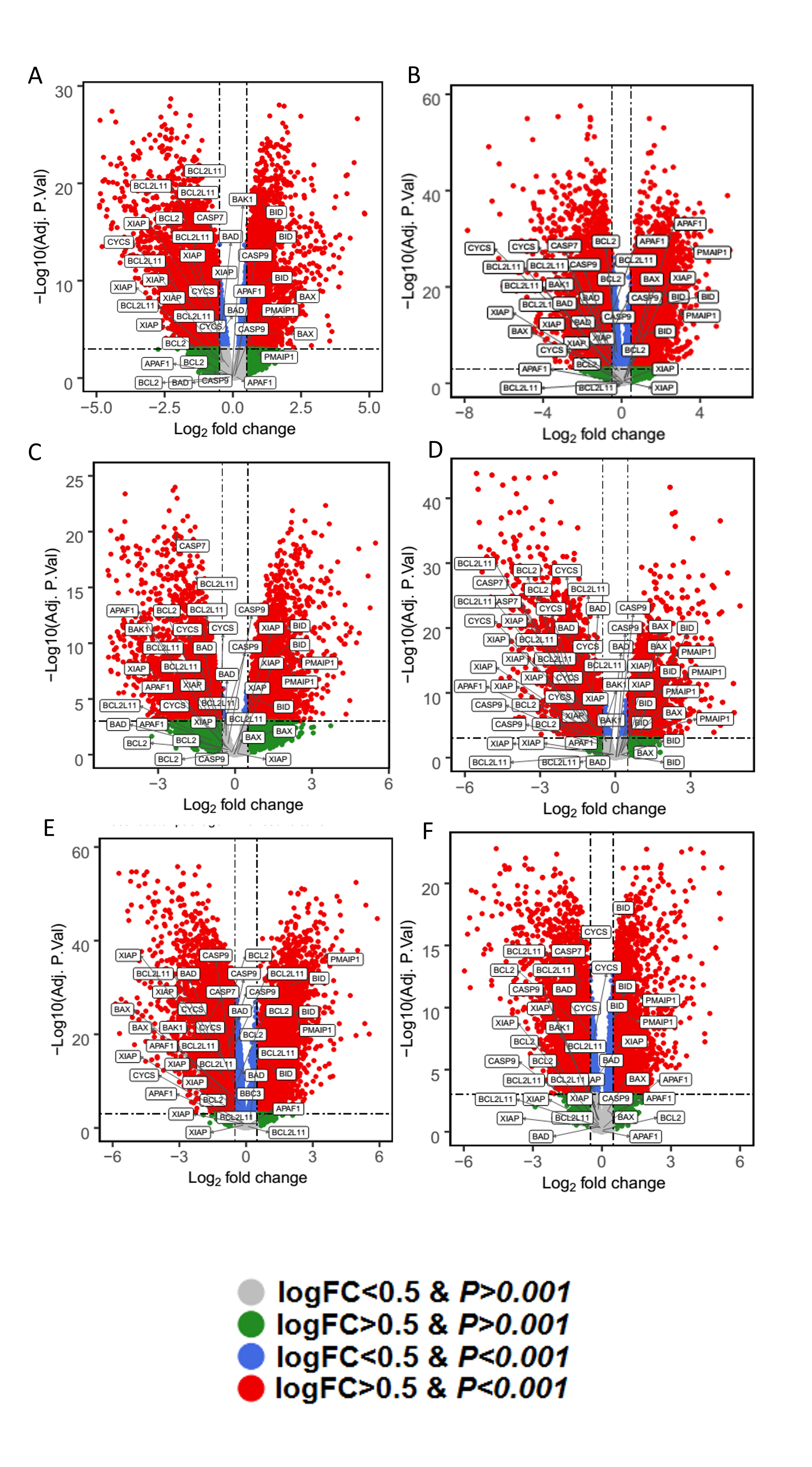

Supplement: Supplementary file 2 — Additional file 2: Figure S1. Volcano plot of differential expressed genes (DEGs) between CRC and normal samples from different GSEs based on adj.p-value and log2 (fold-change) at level of 0.0001 and logFC cutoff of 0.5. Colored dots correspond to individual genes whose expression differences were significant based on both adj.p-value and logFC value (red dots), only p-value (blue dots), only logFC (blue dots), or not significant (green dots) in either. [file 12935_2020_1633_MOESM2_ESM.tif]

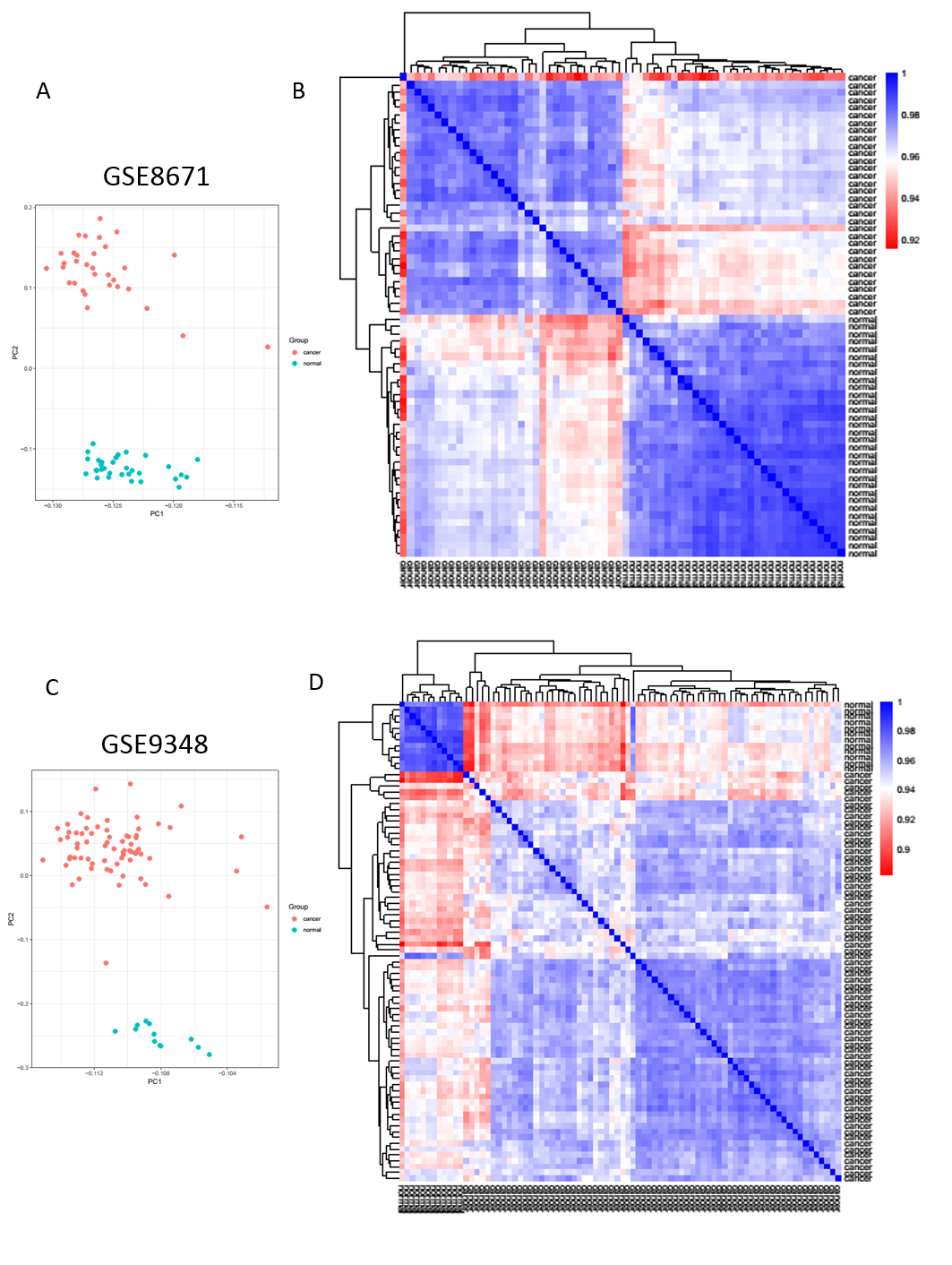

Supplement: Supplementary file 3 — Additional file 3: Figure S2. The quality control results of two dataset GSE8671 and GSE9348 by utilizing PCA and heatmap. [file 12935_2020_1633_MOESM3_ESM.tif]
