# Supplementary material for: Integrative in silico and in vitro transcriptomics analysis revealed new lncRNAs related to intrinsic apoptotic genes in colorectal cancer
Source: Cancer Cell Int. 2020 Nov 10;20:546. doi: 10.1186/s12935-020-01633-w (PMC7653898; doi:10.1186/s12935-020-01633-w)
Supplement: Supplementary file 4 — Additional file 4: Table S2. LncRNAs list with the highest expression correlation with each of the candidate genes. [file 12935_2020_1633_MOESM4_ESM.docx]

Table S2: LncRNAs list with the highest expression correlation with each of the candidate genes

| probe ID | Degree | In Annotation of Manufacturer (NCBI) | hugo name | lncRNA Name |
| --- | --- | --- | --- | --- |
| 1559884_at | 6 | **CDKN2B-AS1** | CDKN2B-AS1 | lnc-MTAP-1 |
| 230991_at | 6 | **LOC102724156** | LOC100653206 | lnc-CDK20-8 |
| 239182_at | 6 | **HAGLR** | HOXD-AS1 | lnc-AC009336.1-2 |
| 1552583_s_at | 6 | **ABCC13** | ABCC13 | lnc-RBM11-5 |
| 233527_at | 6 | **LOC101929340** | LOC100129069 | lnc-NRGN-1 |
| 236001_at | 6 | **LINC00675** | LOC100289255 | lnc-PIRT-1 |
| 237494_at | 5 | **FAM120AOS** | FAM120AOS | lnc-FAM120AOS-4 |
| 222307_at | 5 | **PDCD4-AS1** | LOC282997 | lnc-BBIP1-1 |
